# PERSON-CENTERED CARE: SETTING THE INTERNATIONAL SCENE FROM A CONCEPTUAL PERSPECTIVE

**DOI:** 10.1093/geroni/igad104.0966

**Published:** 2023-12-21

**Authors:** Tonya Roberts, Annica Backman

**Affiliations:** University of Wisconsin, Madison, Wisconsin, United States; Umea University, Umea, Vasterbottens Lan, Sweden

## Abstract

Over 142 million older persons worldwide need assistance with meeting basic needs, often requiring some level of long-term support and services. Person-centered care (PCC) is internationally recognized as an essential strategy for achieving high quality care and quality of life for older adults who need long-term care. Rather than focusing only on disease management or preventing morbidity, PCC results in positive outcomes for older people and their caregivers by focusing care on the whole person in their unique environment and aligning care with what is holistically meaningful to older people. Although PCC has become the gold standard for care of older adults, its widespread implementation has led to theoretical, conceptual, and practical inconsistencies that make it challenging to compare or evaluate its application or effectiveness globally. This is particularly problematic given our understanding of PCC is largely derived from research and practice in western cultures. Despite positive outcomes, the wide spectrum of person-centered interventions introduces variation which has made global comparisons difficult and ability to identify needs for cultural adaptation largely absent. To be able to compare PCC internationally, and reflect on potential needs for cultural adaptation, more conceptual clarity of the concept of PCC, its measurement, and implementation is needed. The purpose of this presentation is to review the use, value, and implementation of PCC concepts in international literature and present possible future directions for extending PCC to be more culturally inclusive for a global older adult population.

